# Twelve Tips for Recognizing and Addressing the Adverse Effects of Medical Education Interventions: Tutorial

**DOI:** 10.2196/89647

**Published:** 2026-05-22

**Authors:** Tadayuki Hashimoto, Mohammad Adrian Hasdianda, Kanapa Kornsawad, Shunsuke Kosugi, Makoto Kikukawa

**Affiliations:** 1 Department of Emergency Medicine, Brigham and Women’s Hospital, 75 Francis St, Boston, MA, 02115, United States, 1 6173720780; 2 Osaka Medical and Pharmaceutical University, Takatsuki, Osaka, Japan; 3 The University of Texas Health Science Center at San Antonio, San Antonio, TX, United States; 4 Department of Medical Education, Faculty of Medical Sciences, Kyushu University, Fukuoka, Japan

**Keywords:** medical education, educational interventions, adverse effects, unintended consequence, educational harm

## Abstract

“First, do no harm” is a fundamental principle in health care, and clinical researchers carefully monitor adverse drug reactions to ensure patient safety. However, educational researchers and clinical educators rarely apply the same level of scrutiny to potential adverse effects arising from their own interventions. This reflects a persistent misconception that educational interventions are inherently harmless, an assumption that warrants critical examination. In this tutorial, we highlight the underrecognized concept of adverse effects in medical education by introducing 12 representative educational adverse effects and offering corresponding tips for mitigating them. These include the Dunning-Kruger effect, in which increased confidence does not align with actual competence; the undermining effect, whereby external rewards reduce intrinsic motivation; spoon feeding that stunts independent learning; cognitive overload resulting from excessive information delivery; patient dehumanization when education prioritizes technical proficiency over empathy; critiques of outcome-based medical education that may overemphasize measurable competencies at the expense of holistic professional development; and the expertise reversal effect, in which instructional strategies beneficial for novices become counterproductive as expertise grows. Additional adverse effects include compromised psychological safety despite formal safeguards, authority and confirmation biases that reinforce outdated practices, developer bias in intervention evaluation, the Hawthorne effect influencing observed behavior, and concerns that overreliance on generative artificial intelligence may hinder the development of critical thinking and metacognitive skills. To better understand the nature of these adverse effects, we categorize them into three overarching domains. Cognitive and psychological adverse effects occur within the learner. Structural and cultural adverse effects result from features of the educational environment. Methodological and evaluative adverse effects arise from how educational interventions are designed and assessed. While these domains overlap, they provide a practical framework for identifying how well-intended educational strategies may lead to harm. Some may argue that these phenomena represent unintended consequences rather than adverse effects. However, the term unintended consequence presumes that sufficient effort was made to anticipate and manage possible effects, an assumption that may not always hold in medical education. We argue that educators and educational researchers should explicitly recognize adverse effects, critically evaluate educational interventions, and adopt mitigation strategies with a level of rigor comparable to that applied in clinical research to better protect learners and improve the quality of medical education.

## Introduction

“First, do no harm” is widely recognized as a fundamental principle in health care [1]. All health care professionals are taught to uphold and practice this principle. In line with it, clinical researchers carefully design and conduct rigorous clinical trials, particularly when introducing new medications. Among these considerations, monitoring for adverse drug reactions is given the highest priority to ensure patient safety [2].

However, do educational researchers and even clinical educators apply the same level of scrutiny to potential adverse effects in their own interventions? The assumption that educational interventions are inherently harmless is a misconception. While often overlooked, certain educational strategies may result in educational adverse effects, an issue that remains underexplored in medical education research. Similar concerns have recently been increasingly recognized in related fields, including psychosocial [3,4] and public health interventions [5].

In this review, we will highlight the underrecognized concept of adverse effects in medical education. To help prevent not only adverse outcomes in educational research but also those that may occur in day-to-day teaching and negatively affect learners, we introduce 12 representative educational adverse effects and offer 12 corresponding tips for mitigating them.

## Tip 1: Confidence Hit the Wall of Ignorance (Dunning-Kruger Effect)

Providing information and training to beginners can enhance their confidence; however, this confidence may not align with actual competence, potentially leading to poor patient outcomes. This mismatch, known as the Dunning-Kruger effect, refers to a cognitive bias whereby individuals with limited expertise overestimate their abilities [6]. For instance, in a study on simulation-based training for joint injections among primary care physicians, those who reported higher confidence after training paradoxically performed worse in real clinical settings. (*r*=−0.253; *P*=.02) [7]. Such overconfidence can impair patient care and hinder proper skill development [8-10].

To mitigate this, educators can begin addressing this issue by acknowledging the presence of overconfidence and focusing on enhancing learners’ metacognitive skills, enabling them to better evaluate their own competence [8]. Researchers, meanwhile, should refrain from relying solely on self-reported confidence as an outcome measure, a practice still common in educational studies [11,12].

## Tip 2: Incentives Killed the Curiosity (Undermining Effect)

External rewards intended to motivate learners can paradoxically diminish their intrinsic motivation, a phenomenon known as the undermining effect [13]. This occurs when external incentives—such as grades or performance-based rewards—overshadow learners’ internal drive to understand or improve. A meta-analysis investigating the impact of rewards on learner motivation found that engagement-, completion-, and performance-contingent rewards significantly reduced intrinsic motivation in free-choice learning tasks (effect sizes –0.40, –0.36, and –0.28, respectively) [14]. In medical education, a realist synthesis examining the impact of assessment on health care learners’ motivation revealed that many evaluation systems encouraged surface-level learning and negatively impacted psychological well-being [15].

One effective way to encourage deeper clinical reasoning is for educators to move beyond standard multiple-choice questions and incorporate patient-centered tasks that require explanation and engagement [15]. These promote deeper engagement and align assessment with meaningful clinical competence. Researchers should also be cautious of the Hawthorne effect, where temporary motivation surges during the study period may obscure the intervention’s true long-term impact [16].

## Tip 3: Teach Learners to Learn, and You Feed Them for a Lifetime (Spoon Feeding)

When learners become accustomed to passively receiving one-way information, so-called spoon feeding, their intellectual and psychological growth can be stunted [17]. Teachers who deliver content without actively involving students risk undermining both independent learning and creativity [18]. Criticisms of this passive approach have persisted in medical education for over a century [19]. The solution is well-known: a shift toward active and self-directed learning [20]. Recent meta-analytic evidence supports this pedagogical transition. For instance, flipped classroom models have been shown to significantly improve both test scores (standardized mean difference 0.481, 95% CI 0.214-0.748) and skills performance (standardized mean difference 0.660, 95% CI 0.312-1.008) compared to traditional lectures [21].

However, the true challenge lies not in recognizing the problem, but in implementing meaningful cultural and institutional change [22,23]. At the individual level, educators should adopt active learning strategies within their own teaching contexts. At the institutional level, such as in medical schools, researchers should investigate how best to scale these pedagogical shifts—particularly by examining why adaptive learning models like problem-based learning, despite their theoretical appeal, have struggled in certain settings [24].

## Tip 4: More Is Too Much (Cognitive Overload)

Educators often assume that more teaching leads to more learning. However, excessive delivery of information can overwhelm learners, sometimes resulting in lower retention than if less had been taught. This phenomenon is explained by cognitive load theory, which holds that working memory has a limited capacity to process information at any given time [25]. For example, in one study, intensive care unit attendings gave lectures with three key take-home messages. When 367 residents were later surveyed, two-thirds could recall only one message [26]. This illustrates the limits of cognitive processing under high load.

To mitigate this, educators should embrace the “less is more” principle in medical education, focusing not merely on the quantity of information but on managing the cognitive load imposed on learners [27]. Researchers, in turn, should explore strategies to optimize working memory use and reduce extraneous load in educational interventions [25].

## Tip 5: The Patient Who Wasn’t There (Patient Dehumanization)

Patient dehumanization occurs when learners begin to perceive patients not as individuals with emotions and unique needs, but merely as clinical cases—or worse, as objects to be examined and managed. This shift can stem from multiple factors, including stress, rigid hierarchies, and an educational focus that prioritizes technical proficiency over empathy [28]. Although simulation-based training is valuable for skill acquisition [29], it may inadvertently contribute to this issue. In one study comparing two groups of medical students—one trained primarily through simulation and the other through bedside clinical clerkships—those in the simulation group scored significantly lower on measures of compassion (*P*=.02) [30].

To address this, educators must acknowledge the power dynamics inherent in clinical settings and remind learners that patients are not objective entities but sentient individuals with emotional experiences. This aligns with the concept of the iPatient—a term popularized by Dr Verghese [31]—which highlights how increasing reliance on electronic health records can shift attention away from real patients toward their digital representations. Researchers should critically evaluate educational interventions, particularly simulation-based training, for potential adverse effects such as dehumanization. This is especially important given that even existing reporting guidelines for simulation research rarely address this humanistic dimension [32].

## Tip 6: In Objectivity We Trust (Critiques of Outcome-Based Medical Education)

Science prioritizes objectivity to ensure reproducibility. A prominent example of this in medical education is outcome-based medical education (OBME), which began to gain traction around the year 2000 and has since been implemented globally. This trend has mirrored the concept of entrustable professional activities as its most advanced form [33]. However, the emphasis on objectively measuring learner competence has also drawn criticism. Specifically, the rigid focus on predefined outcomes can oversimplify the nuanced, context-dependent skills required for clinical practice, reducing them to narrowly defined competencies [34]. For example, surgical safety checklists, widely adopted to enhance patient safety, emphasize standardized tasks like the “sign-out” phase. One study found that, following the implementation of a surgical safety program that included an educational intervention, sign-out completion improved from 79.3% to 94.5% (*P*<.0001), yet clinical incidents rose from 0.13% to 0.25% [35]. This raises concerns that an overemphasis on measurable behaviors may obscure more complex, less visible dimensions of patient safety.

OBME may also constrain educators’ creativity by limiting flexibility in teaching [36]. Rather than focusing solely on quantifiable outcomes, educators should preserve space to address essential but less easily measured aspects of training. From a research standpoint, there remains limited evidence demonstrating OBME’s long-term effectiveness. While funding and logistical constraints often hinder the assessment of patient-level outcomes, efforts should be made to evaluate the broader impact of OBME on clinical care [34].

## Tip 7: A Farewell to Scaffolds (Expertise Reversal Effect)

As learners gain expertise, their cognitive load decreases, enabling them to benefit more from less structured, problem solving–based learning environments. Instructional methods that were once helpful for novices may become less effective—or even counterproductive—for more advanced learners. This phenomenon is known as the expertise reversal effect [37]. Multiple studies have demonstrated this effect. For example, novices benefited from additional explanatory text accompanying animations, while experts performed better without it [38]. Similarly, presenting correct examples before erroneous ones reduced mental load and improved learning outcomes for novices but had little impact on expert learners [39].

To address this, educators should not avoid categorizing learners based solely on their year or generation [40]; instead, they should assess learners’ cognitive developmental stage, such as those described in the RIME (reporter to interpreter to manager to educator) model [41]. Future research should explore adaptive learning systems that tailor instruction to learners’ expertise in real time. For example, intelligent tutoring systems and simulation platforms could adjust case complexity or the level of guidance based on a trainee’s performance and prior knowledge. Evaluating the effectiveness of such personalized, just-in-time instructional strategies could help translate the principles of the expertise reversal effect into broader educational practice.

## Tip 8: Let Me Be Wrong (Psychological Safety)

Psychological safety is increasingly recognized as a foundational element of effective learning in medical education. It refers to the belief that one can take interpersonal risks—such as offering questions, admitting uncertainty, or sharing ideas—without fear of embarrassment, punishment, or negative judgment [42,43]. However, even when educators explicitly assure students that these will not impact their evaluations, learners may still tailor their entries to highlight only performance in “safe” or favorable contexts, fearing judgment nonetheless [42,43]. For example, in one study, students were asked to submit reflective portfolios, and they selectively recorded experiences where they performed well, rather than those that truly challenged them [44]. This demonstrates how psychological safety can be compromised despite well-intentioned safeguards. Another example of this paradox can be seen in a study where an asynchronous learning platform was developed to allow residents to reflect at their own convenience. Although residents reported high satisfaction with the perceived educational value, the ability to post during late-night hours unintentionally increased their workload, with many reflections being submitted after midnight [45].

To mitigate this, educators must actively cultivate psychologically safe learning environments, acknowledging that, due to hierarchical structures in clinical education, the primary responsibility lies with supervisors [42,43]. Researchers should also prioritize the development and evaluation of interventions aimed at enhancing psychological safety, as prospective studies in this area remain relatively limited [46].

## Tip 9: Old Ways Never Die (Authority Bias)

As educators, we must recognize that learners are particularly vulnerable to cognitive biases such as authority bias—the tendency to unquestioningly accept information from perceived experts [47]—and confirmation bias—the tendency to disregard evidence that contradicts pre-existing beliefs [48,49]. When trusted educators convey outdated or incorrect knowledge, these biases can reinforce misinformation and hinder evidence-based practice [50]. For example, the concepts behind initiatives such as “Things We Do for No Reason” and the campaign of “Choosing Wisely” illustrate how certain clinical practices persist despite lacking evidence, often due to tradition and uncritical deference to authority [51,52].

To mitigate these biases, educators need to go beyond delivering static content and prioritize the development of learners’ critical appraisal skills and information literacy [53]. Researchers likewise bear responsibility and should avoid perpetuating discredited concepts, such as the widely debunked theory of learning styles [54].

## Tip 10: Developer’s Strangelove (Experimenter Effect and IKEA Effect)

Educational interventions and curricula are often developed by the same individuals who evaluate them. This dual role can introduce unconscious biases, such as the experimenter effect—where researcher expectations unintentionally influence outcomes [55]—and the IKEA effect—the tendency to overvalue one’s own creations [56]. Similar concerns have been raised in pharmaceutical research, where criticism arises when drug developers lead their own clinical trials [57]. Yet in educational research, it is still common for developers to assess the effectiveness of their own interventions. In such cases, researchers’ expectations can shape both the design and interpretation of outcomes, mirroring biases observed in behavioral studies [58].

To address these, educators and researchers should exercise caution when evaluating their own educational products. The involvement of independent evaluators can enhance the objectivity of their intervention [29]. In educational intervention research, adhering to reporting guidelines such as GREET (Guidelines for Reporting Educational Intervention Trials) [59] and relevant educational reporting guidelines [60] can not only ensure transparency in study design and reporting but also minimize potential bias introduced by developers.

## Tip 11: Learners Are Watching You (Hawthorne Effect)

Direct observation is widely regarded as one of the most effective assessment tools in medical education [61]. Training programs and specialties have now defined required competencies, subcompetencies, developmental milestones, performance levels, and entrustable professional activities that can be directly observed and assessed [33]. However, the act of observation itself may influence behavior, a phenomenon known as the Hawthorne effect [16,62]. For example, when unannounced standardized patients were used to assess health care teams, some patient safety measures were found to be alarmingly deficient: only 15% of patients were screened for depression using the 2-item Patient Health Questionnaire, and 5% of clinicians performed hand hygiene before examinations [63].

Educators should be aware of the potential influence of observation and consider incorporating unscheduled direct observations or unannounced standardized patients as part of a broader, multimethod evaluation strategy. Researchers, in turn, should recognize that although behavioral change—as described in Kirkpatrick’s third level of evaluation—is valuable, the ultimate goal remains to measure outcomes. Given the limited empirical evidence for the Hawthorne effect, further investigation into its actual impact on educational evaluations may still be warranted [64].

## Tip 12: Do Learners Dream of Thinking Skills? (Concerns of Generative Artificial Intelligence)

The advancement of artificial intelligence (AI) has been remarkable and is undeniably reshaping the educational landscape. However, concerns have emerged that overreliance on generative AI (GenAI) may reduce meaningful learning opportunities. A 2024 systematic review suggested that excessive dependence on GenAI may hinder the development of key cognitive skills, including critical thinking, creativity, problem-solving, self-regulation, and metacognition [65]. Moreover, 83% of faculty members expressed concern that GenAI could reduce or eliminate essential human interactions in feedback and instructional processes [66].

To address these issues, educators should use GenAI with a clear understanding of its limitations, particularly about factual inaccuracies, lack of medical expertise, ethical considerations, and financial costs [67]. Recent frameworks emphasize the importance of clinical supervision strategies when learners use AI tools [68]. These include establishing clear guidelines for appropriate AI use, implementing structured supervision processes to monitor AI-assisted clinical decisions, and fostering reflective practices that help learners critically evaluate AI-generated outputs rather than accepting them uncritically. Researchers should prioritize investigations into the ethical implications of GenAI in medical education and its potential long-term effects on learner development.

## Conclusion

To better understand the nature of adverse effects in medical education, we categorized the 12 identified examples into three overarching domains. Cognitive and psychological adverse effects occur within the learner, including overconfidence, reduced intrinsic motivation, cognitive overload, loss of autonomy, compromised psychological safety, and diminished learning from overly structured support as expertise grows. Structural and cultural adverse effects result from features of the educational environment, such as rigid outcome-based frameworks, authority bias, practices that may dehumanize patients or hinder empathy, and overreliance on GenAI tools. Methodological and evaluative adverse effects arise from how educational interventions are designed or assessed, including biases introduced by developer-led evaluations and the Hawthorne effect. While these categories may overlap, they offer a practical framework to identify how educational strategies—even well-intended ones—may lead to unintended harm.

Some may argue that these 12 points are not truly adverse effects but rather unintended consequences. However, we would like to challenge whether the term unintended consequence is sufficient or even appropriate in this context. The notion of an unintended consequence implies that there was a deliberate and thoughtful attempt to intend an outcome in the first place. In other words, it presumes that sufficient effort was made to anticipate and manage possible effects, something that may not always be the case in medical education. We, educators and educational researchers, need these efforts more.

## Abbreviations

AI: artificial intelligence
GenAI: generative artificial intelligence
GREET: Guidelines for Reporting Educational Intervention Trials
OBME: outcome-based medical education
RIME: reporter to interpreter to manager to educator
